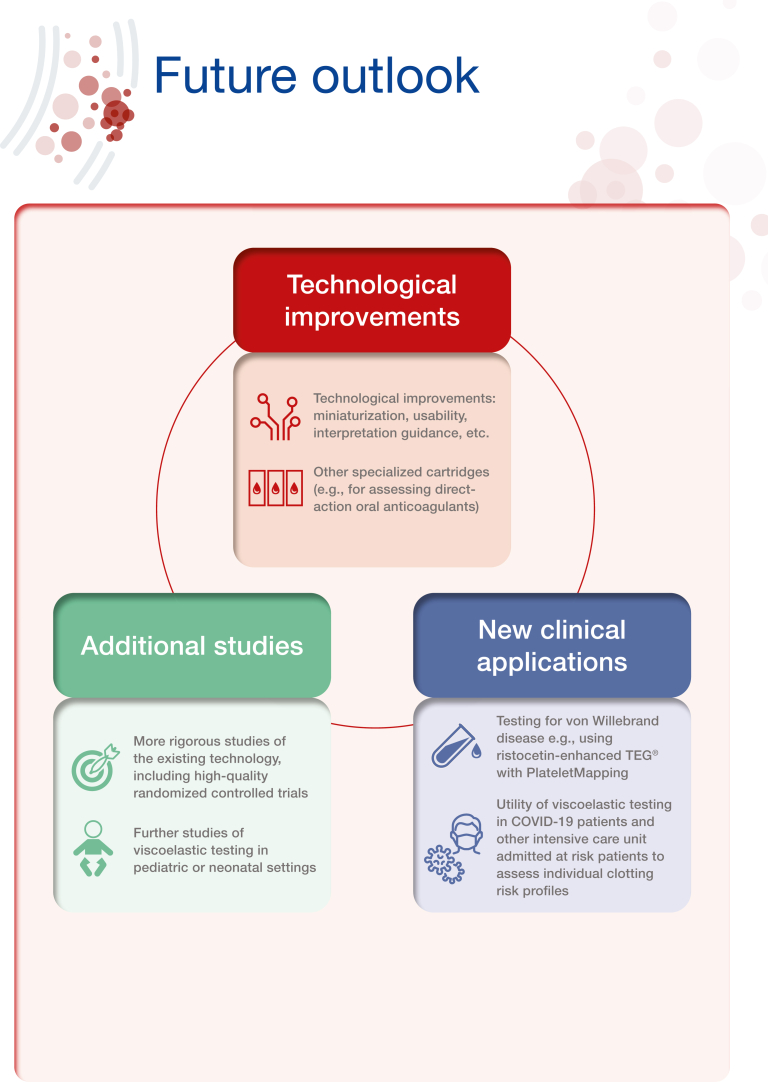# Viscoelastic testing: an illustrated review of technology and clinical applications

**DOI:** 10.1016/j.rpth.2022.100031

**Published:** 2022-12-27

**Authors:** Jan Hartmann, Daniela Hermelin, Jerrold H. Levy

**Affiliations:** 1 Haemonetics Corporation, Boston, Massachusetts, USA; 2 Department of Pathology Saint Louis University School of Medicine, Saint Louis, Missouri, USA; 3 Medical Affairs, ImpactLife, Davenport, Iowa, USA; 4 Duke University Medical Center, Durham, North Carolina, USA

**Keywords:** blood coagulation, clinical applications, fibrinolysis, guidelines, hemostasis, ROTEM, TEG, thromboelastography, thromboelastometry, viscoelastic testing

## Abstract

Viscoelastic testing (VET), including thromboelastography and thromboelastometry, provides a rapid and comprehensive picture of whole blood coagulation dynamics and hemostasis that can be reviewed and evaluated at the point-of-care. This technology is over 50 years old; however, over the past few years, there has been a significant increase in research examining the use of VET. Best practice guidelines for the use of VET exist in both the United States and Europe, particularly for elective cardiac surgery, although recommendations for implementation are somewhat limited in some clinical areas by the lack of studies constituting high-grade evidence. Other challenges to implementation surround validation of the technology in some care settings as well as lack of training. Nevertheless, there is a wide range of potential clinical applications, such as treating coagulopathies in liver disease and transplant surgery, critical care, as well as within obstetrical hemorrhage. In this illustrated review, we provide an overview of viscoelastic testing technology (also called viscoelastic hemostatic assays) and describe how the assays can be used to provide a broad overview of hemostasis from clot formation to clot lysis, while highlighting the contribution of coagulation factors and platelets. We then summarize the major clinical applications for viscoelastic testing, including more recent applications, such as in COVID-19. Each section describes the clinical context, and key publications, followed by a representative algorithm and key guidelines

## Essentials

• Viscoelastic testing (VET) provides a full hemostasis overview from a patient whole blood sample.
• VET can be rapidly reviewed and assessed at the point-of-care and acted upon in real-time.
• Clinical use cases area established in trauma, cardiovascular surgery, and liver transplant areas.
• More high-quality data is needed to identify and further establish clinical benefit areas with VET.

**Figure:**
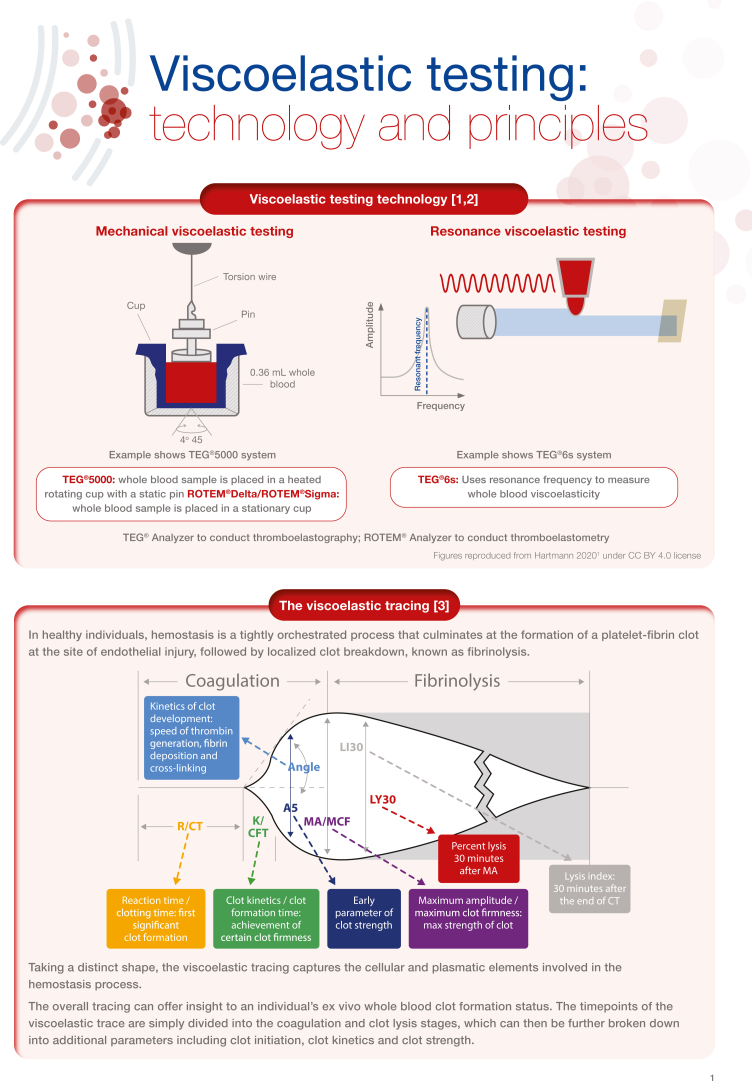

**Figure:**
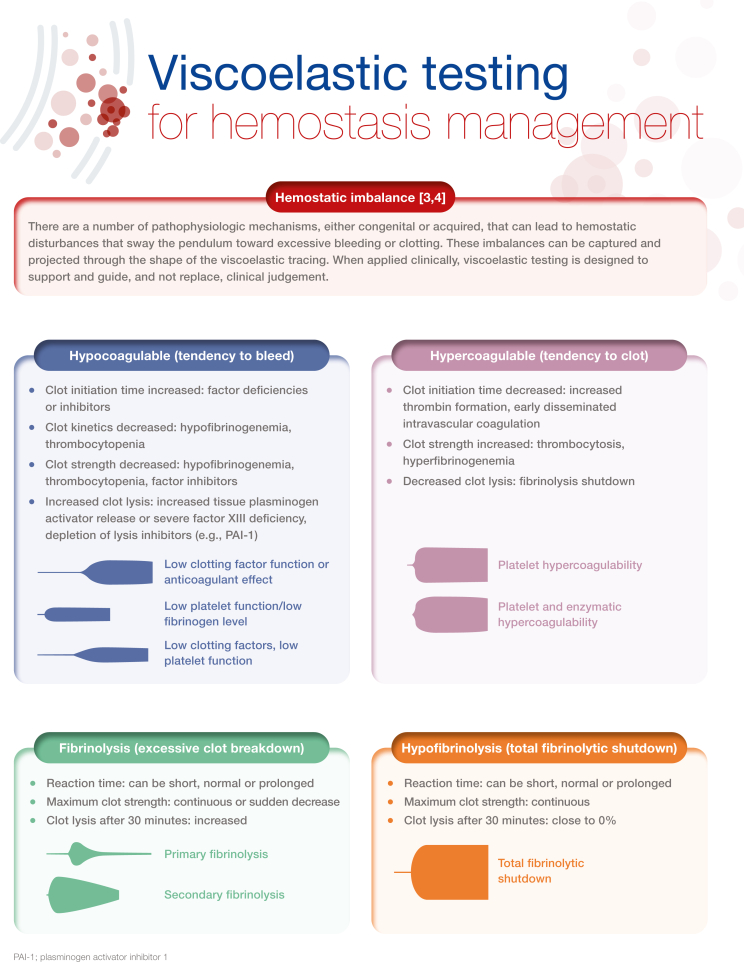

**Figure:**
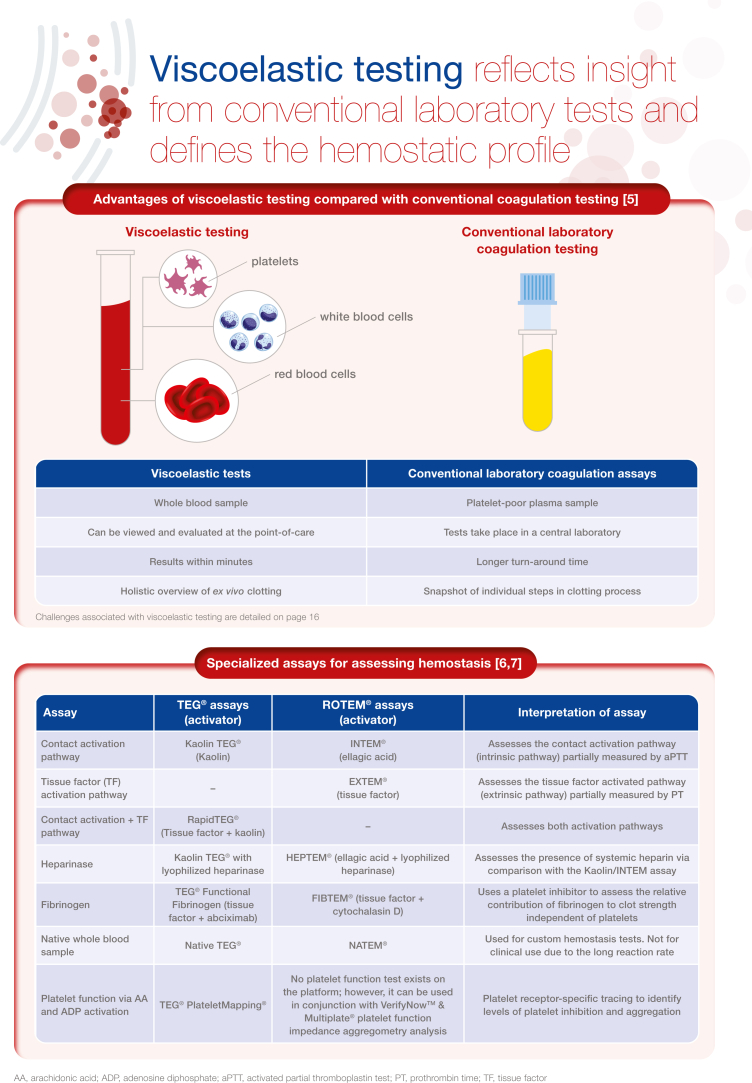

**Figure:**
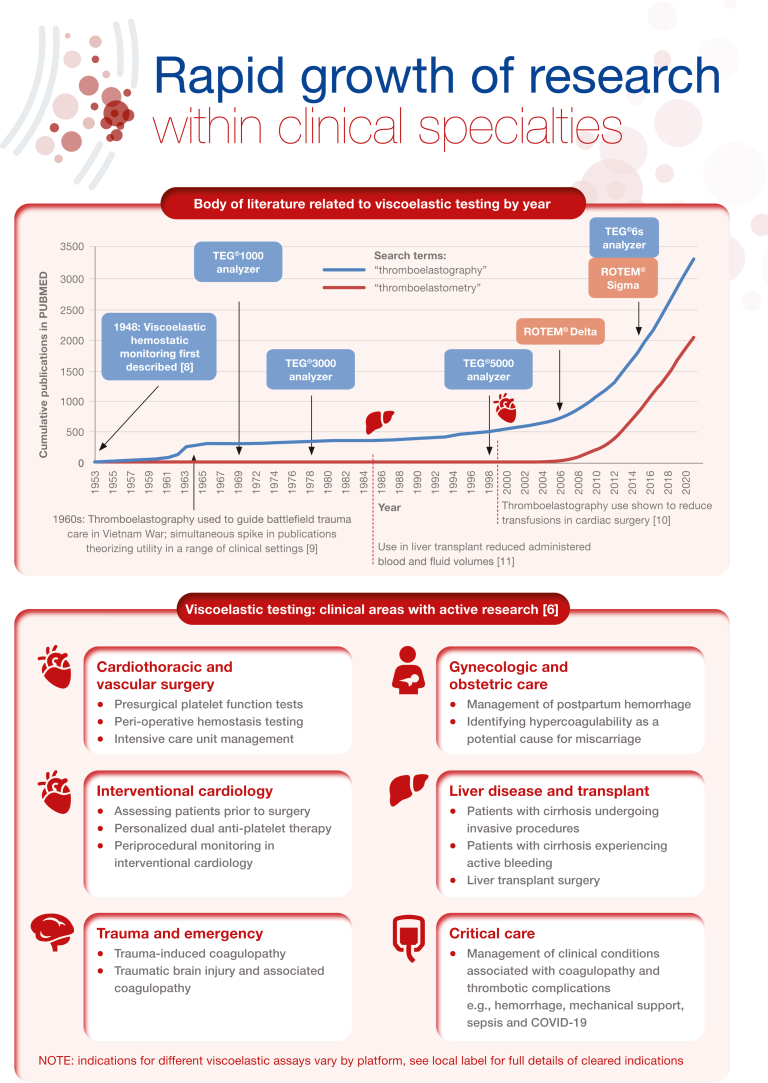

**Figure:**
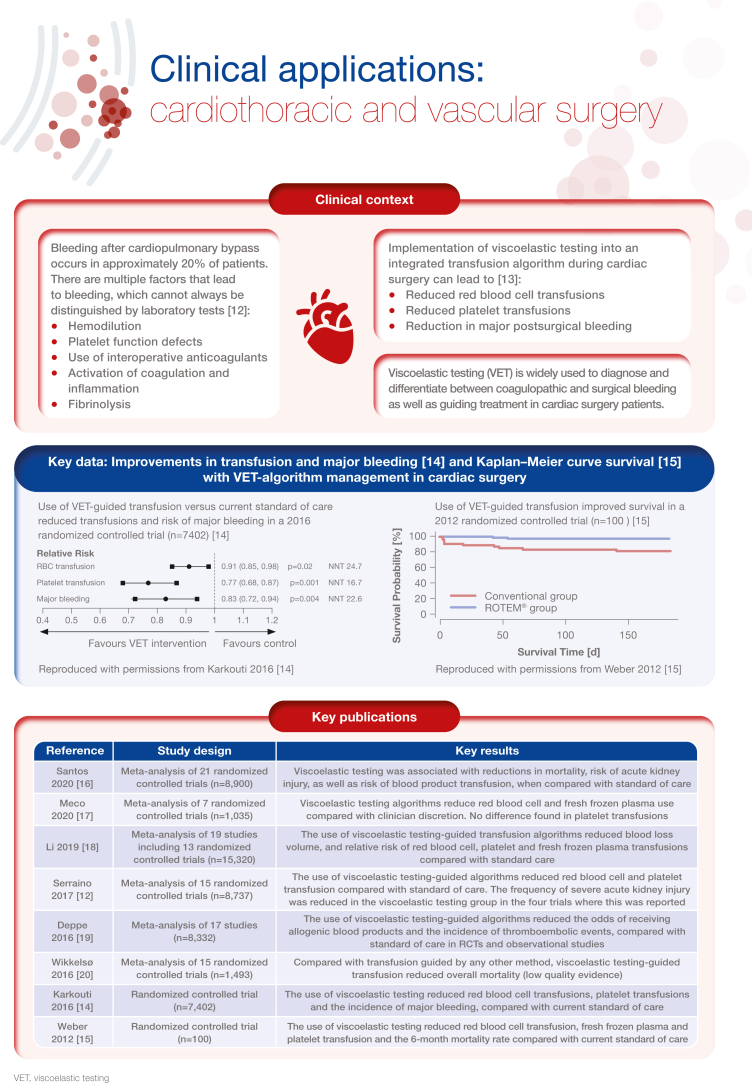

**Figure:**
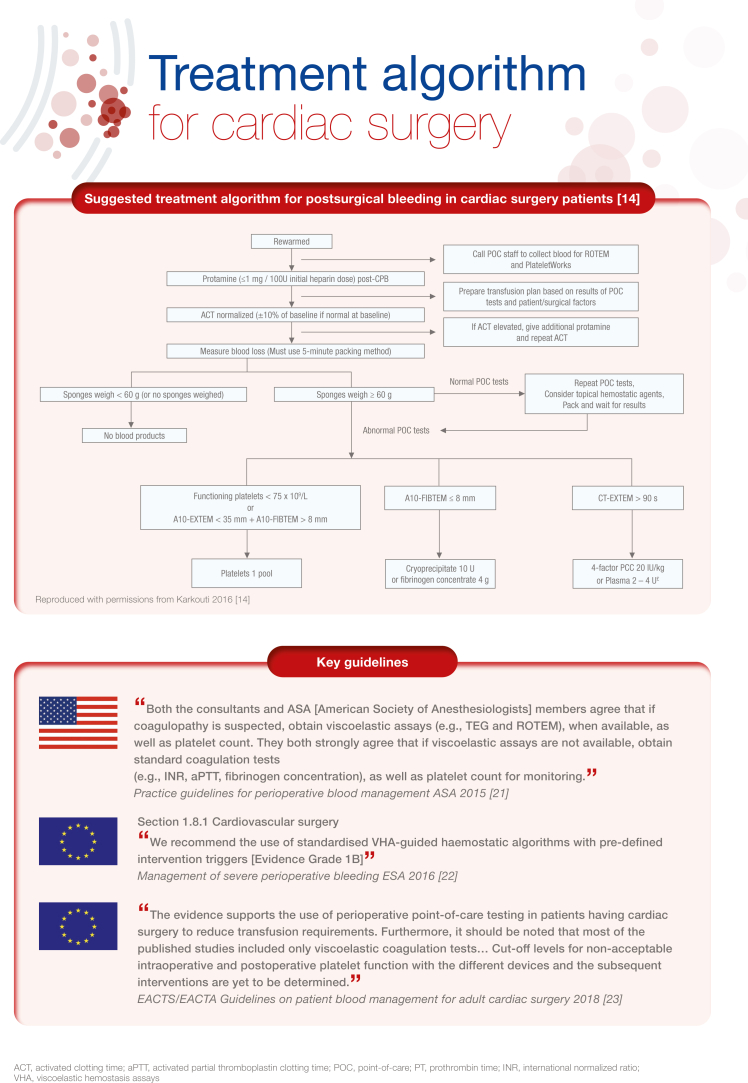

**Figure:**
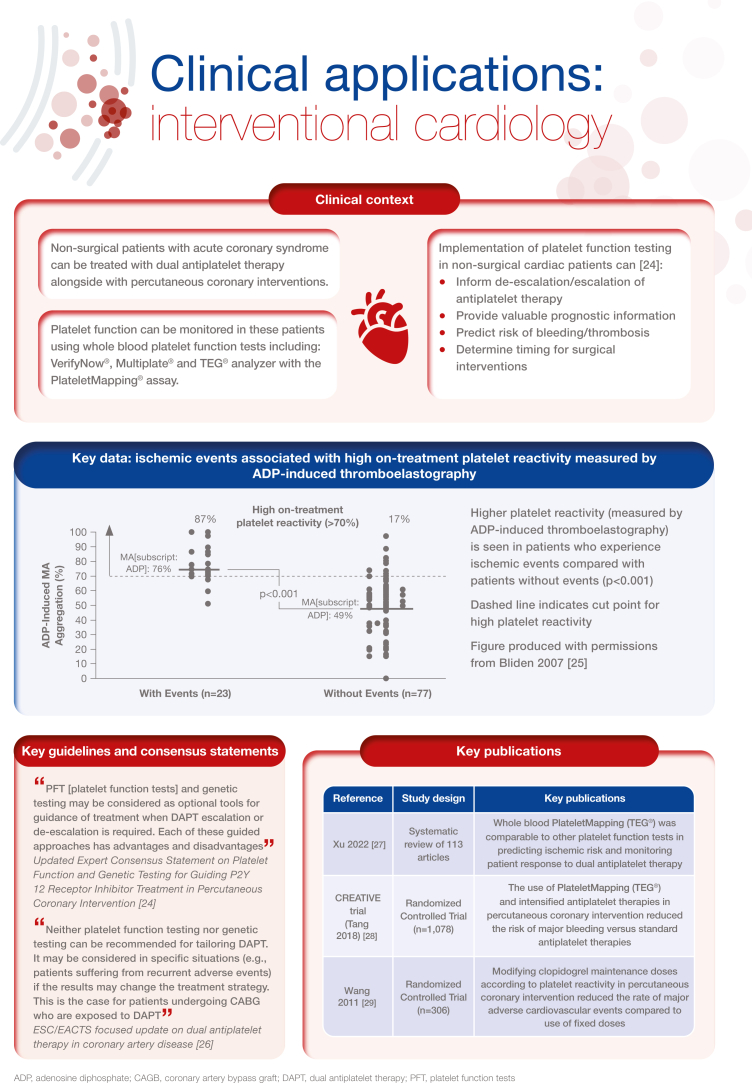

**Figure:**
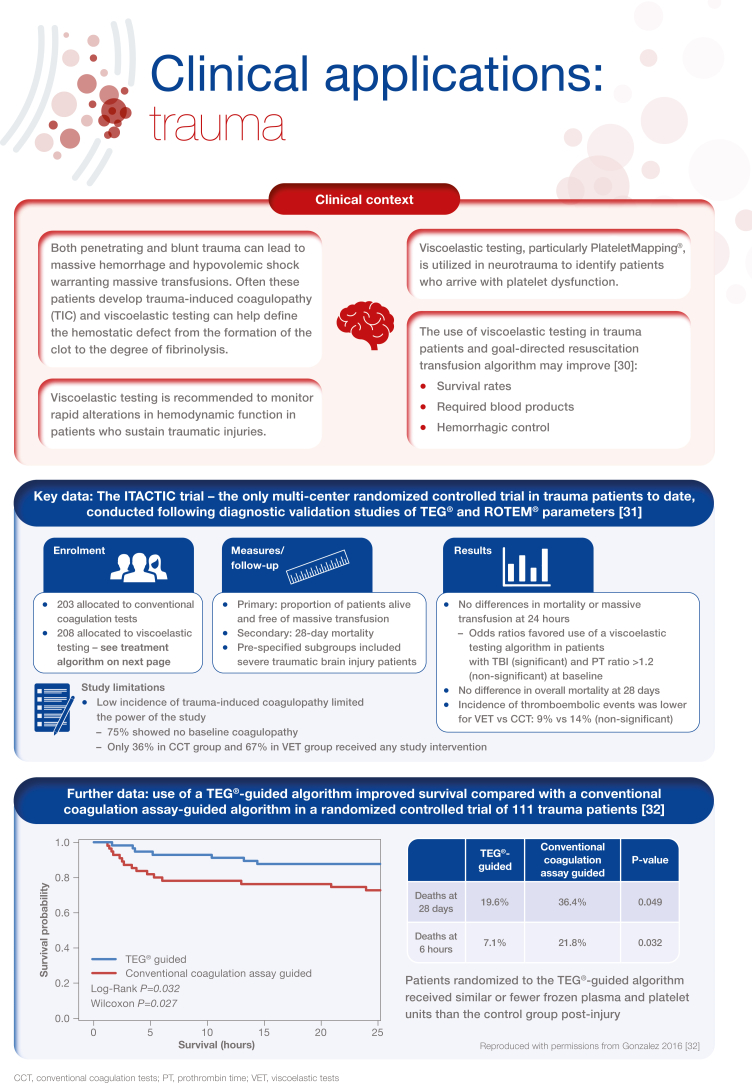

**Figure:**
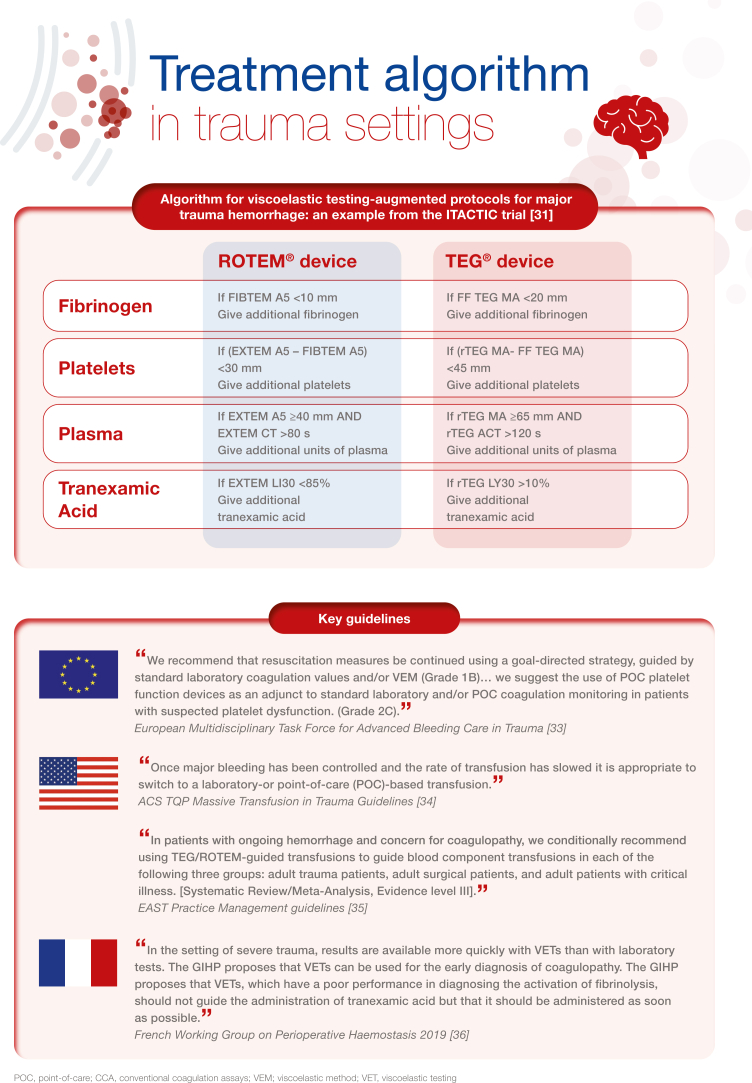

**Figure:**
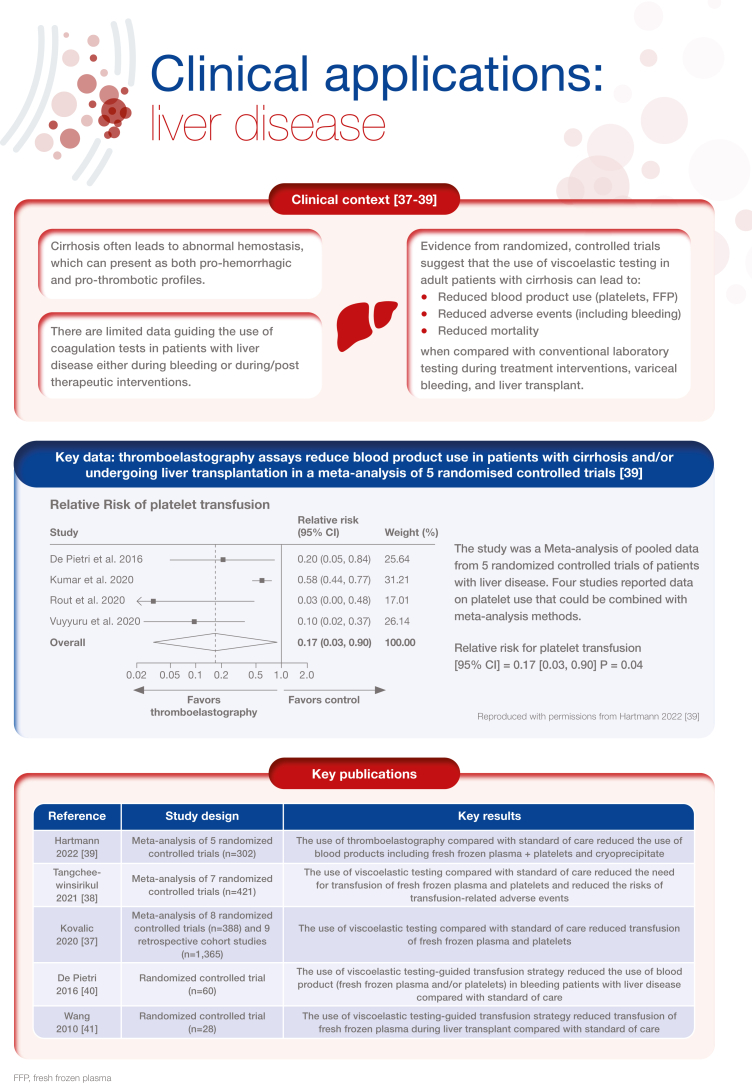

**Figure:**
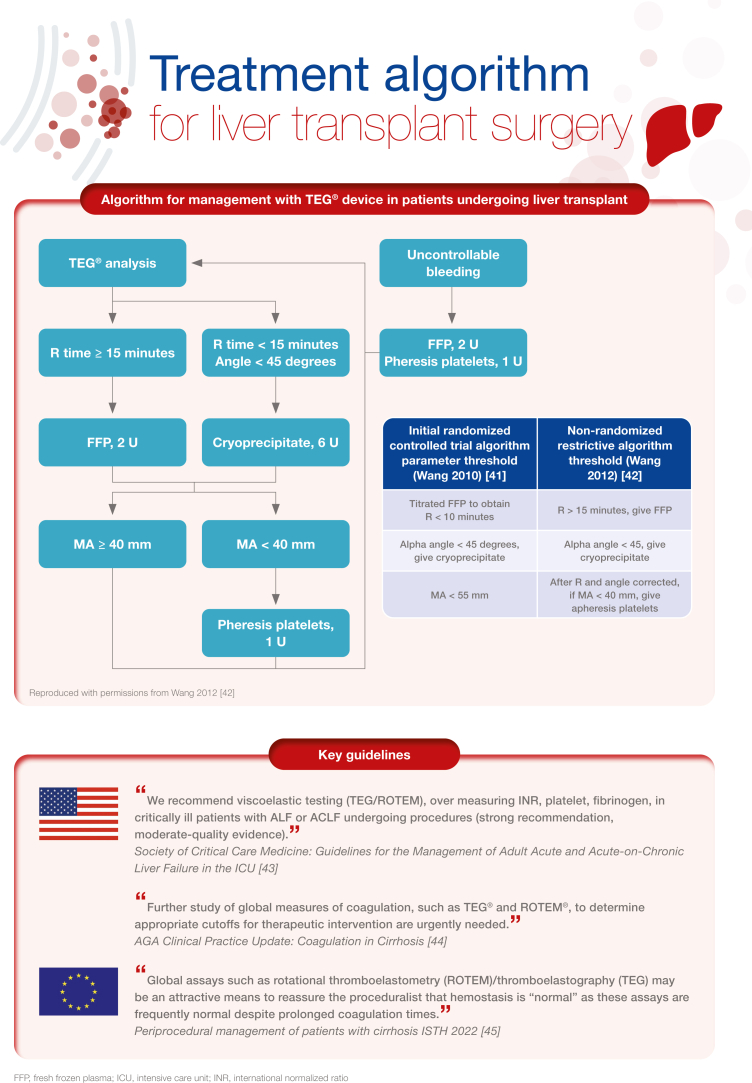

**Figure:**
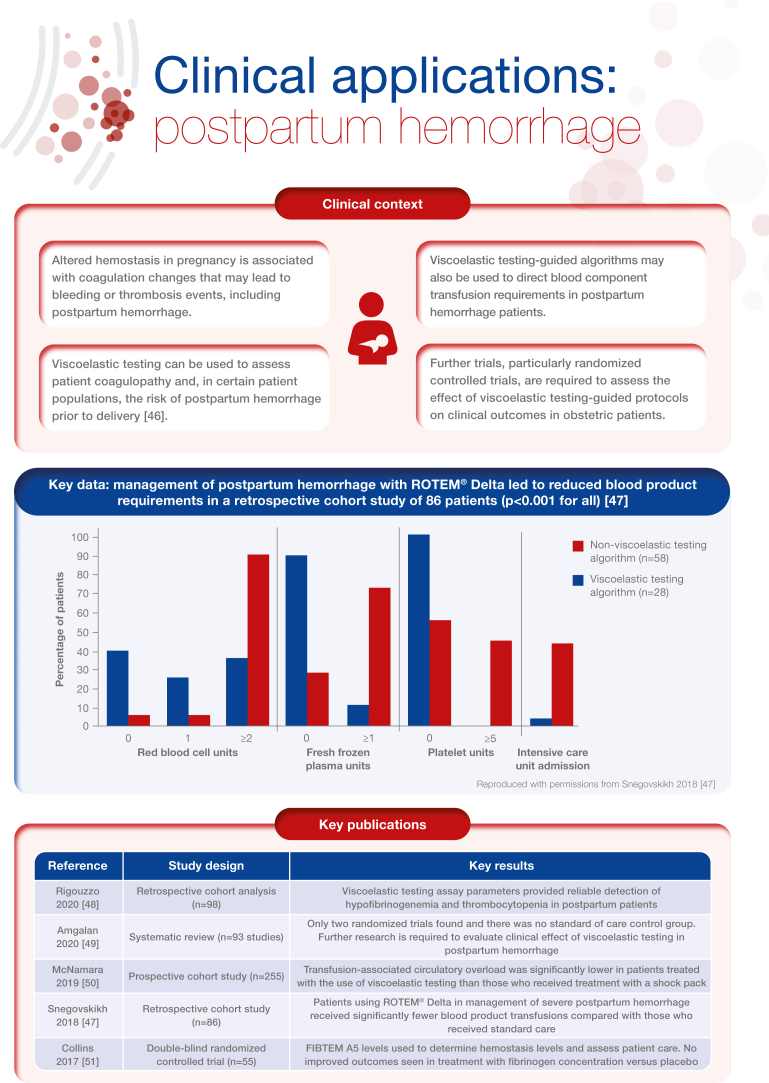

**Figure:**
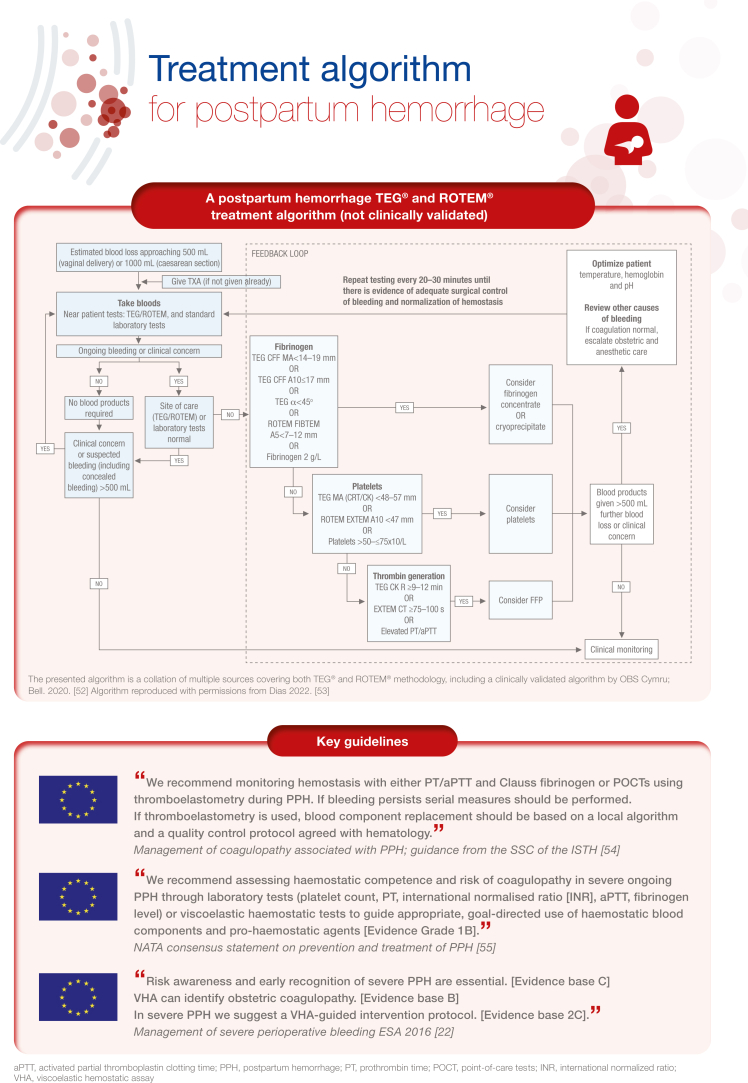

**Figure:**
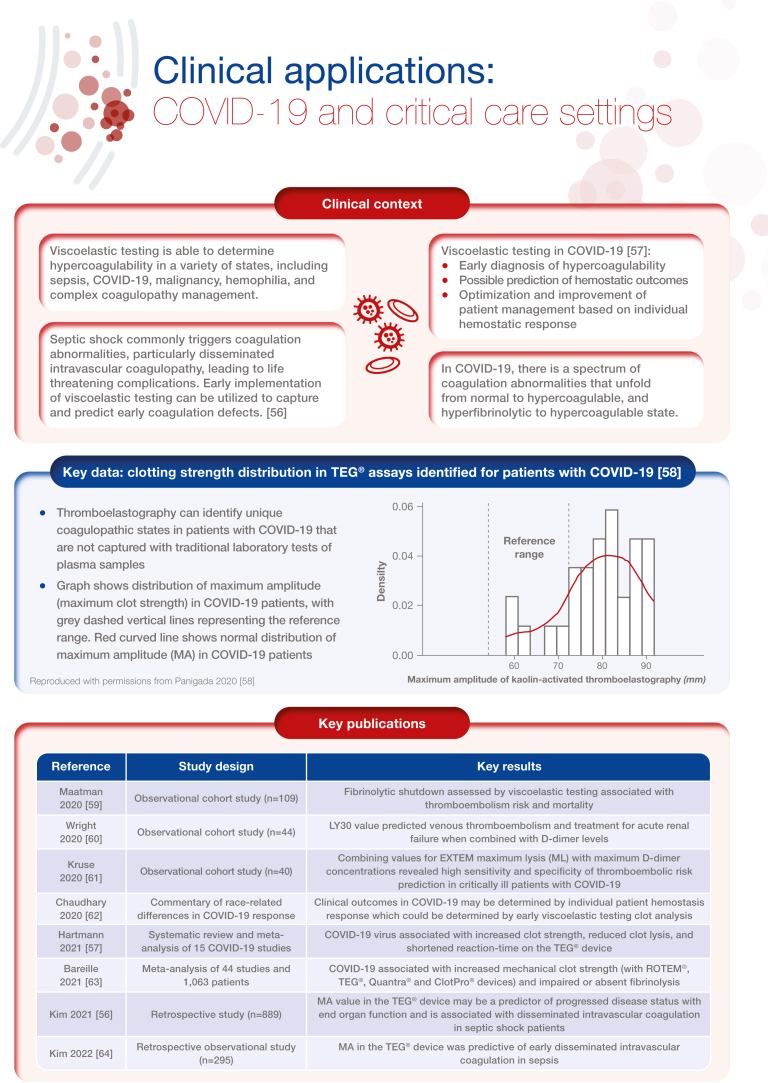

**Figure:**
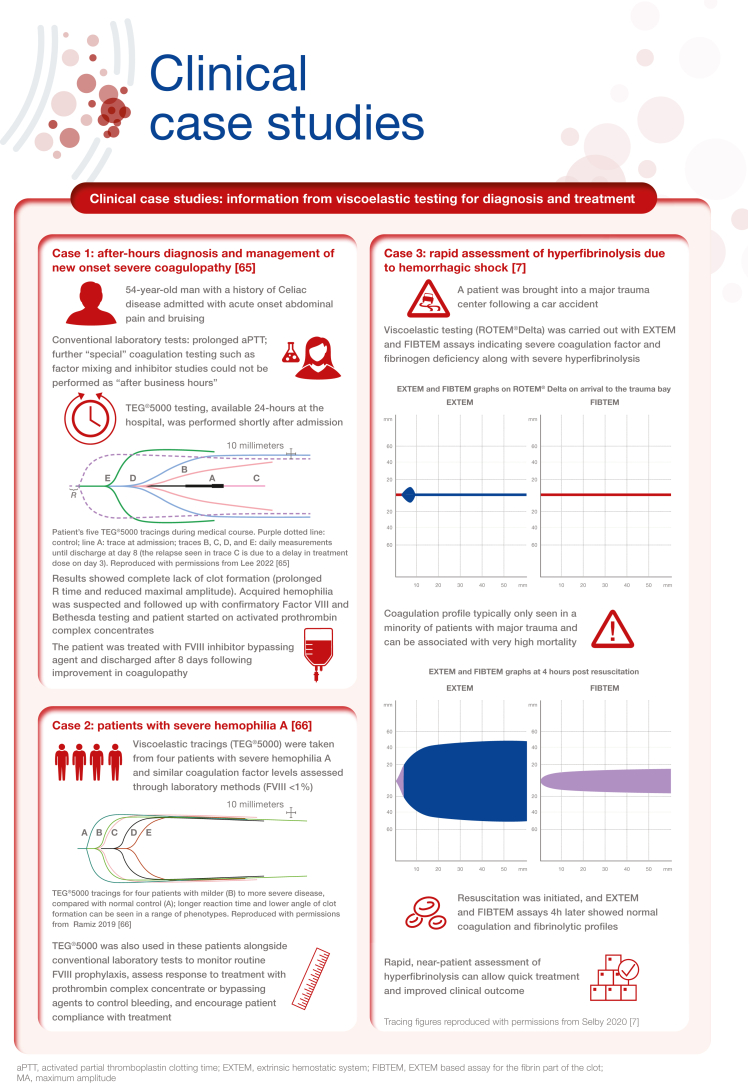

**Figure:**
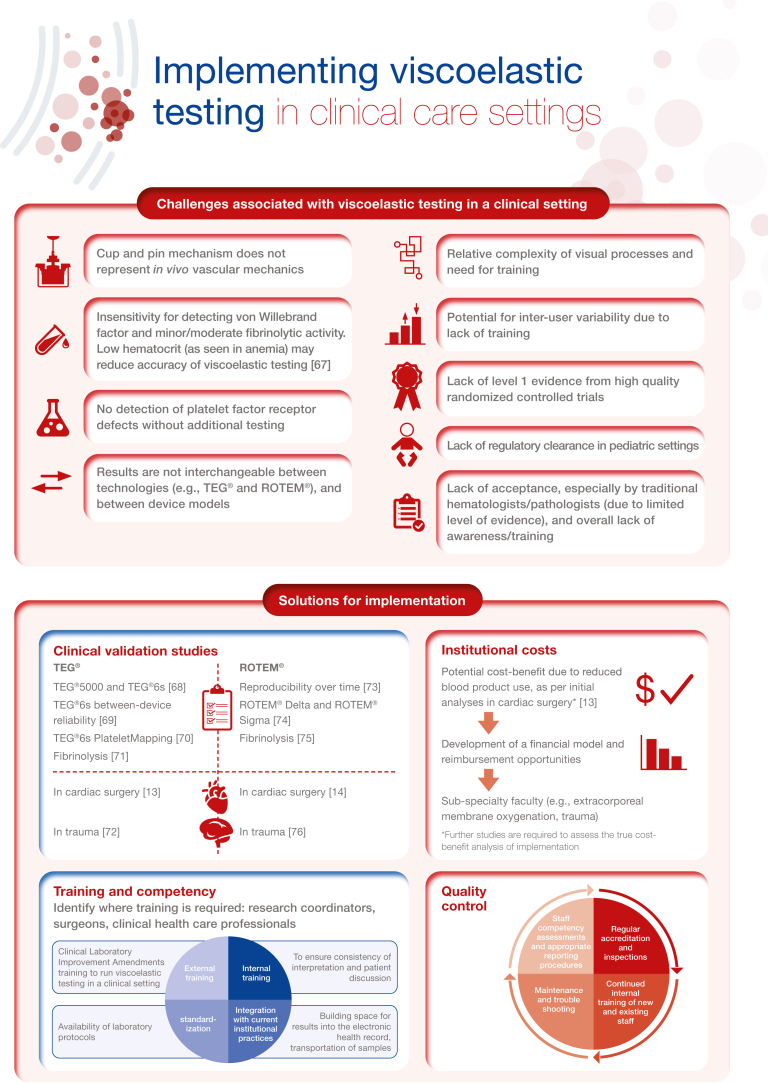

**Figure:**